# The effect of midwifery students' changing life conditions and e-learning experiences on the state of their anxiety and hopelessness during the Covid-19 pandemic

**DOI:** 10.4314/ahs.v24i1.10

**Published:** 2024-03

**Authors:** Mesude Uluşen, Filiz Aslantekin Özçoban, Elif Çilesiz

**Affiliations:** 1 Amasya University, Faculty of Health Sciences, Department of Midwifery, İpekköy, Campus, Amasya/Turkey; 2 Balikesir University, Faculty of Health Sciences, Department of Midwifery, Çagiş Campus, 10100 Balikesir/ Turkey

**Keywords:** Midwife, anxiety, pandemics

## Abstract

**Background:**

Giving the existing formal education through the internet without a planned transition to distance education negatively affected the learning processes of the students.

**Objectives:**

The study aims to identify the midwifery students' changing life conditions and e- learning experiences on the state of their anxiety and hopelessness during the Covid-19 pandemic.

**Methods:**

Designed as cross-sectional research, the study was performed with the participation of 1,296 midwifery undergraduate students. The survey form comprised of questions that explored the students' demographic characteristics, life conditions changing during the Covid-19 pandemic and distance education experiences, the Generalized Anxiety Disorder Scale-7, and the Beck Hopelessness Scale were used in the data collection.

**Findings:**

It was discerned that, of the participant midwifery students, 55.2% spent the period of the pandemic in the province center, 51.7% used smartphones to have access to the distance education, 50.3% had trouble in following up the courses due to the limited computer and internet access, 63.5% failed to follow up the courses because of the infrastructure problems related to the distance education. It was found that, of the participant midwifery students, 54.6% exhibited anxiety symptoms and 26.3% had hopelessness symptoms. It was identified that having trouble in following up the courses increased anxiety by 1.438 times (CI:1.103–1.875) and hopelessness by 1.980 times (CI:1.459–2.687), having tensions in the family relations increased anxiety by 2.362 times (CI:1.780–3.134) and hopelessness by 1.789 times (CI:1.235–2.594), and having psychological support for anxiety and worry increased anxiety by 2.914 times (CI:2.208-3.8477) and hopelessness by 1.875 times (CI:1.083-3.247). It was ascertained that hopelessness increased anxiety by 2.878 times (CI:2.075-3.991) whilst anxiety increased hopelessness by 2.755 times (CI:1.985–3.823) (p<0.05).

**Conclusion:**

As well as the Covid-19 pandemic, health, social life, and economic changes; the digital separation that accompanies distance education also affects the students' mental health. Solving the technical problems experienced in distance education, facilitating the follow-up of the courses, and equipping the midwifery students with problem-solving and coping skills will be useful for reducing the midwifery students' hopelessness and anxiety levels.

## Background

After Covid-19 emerged in December 2019, it spread rapidly and turned into a global pandemic^1^,^2^. The pandemic globally affected the economy, social life, and education, in particular, health^2^,^3^. Due to the pandemic, China, the USA, Italy, Spain, France, Korea, Turkey, Germany, and several other countries had to discontinue face-to-face education and instruction^4^. Nearly 1.6 billion students in more than 190 countries were affected by the pandemic^5^. As the public health measures, restrictive precautions were taken all across the world, and face-to-face education was replaced by distance education^6^-^8^. This process posed serious difficulties as the students and instructors were unaccustomed to distance education and had no experience in it, the current curriculum were based on face-to-face education, there were technical infrastructure problems, and the students had limited computer and internet access^6^,^8^,^9^. It is anticipated that this education style which is hastily created and applied by giving the existing formal education through the internet without a planned transition to distance education will create gaps and have negative effects^6^,^10^,^11^. Particularly the curricula which contain high-level interactive experiences such as the clinical practices and laboratories are quite disadvantaged for the implementation of distance education method^6^,^12^. The fast and dramatic changes accompanying the process of pandemic led also to the interruption of midwifery education, and hence, midwifery education has been suspended in the countries where it was not integrated into the hospitals (Japan, Turkey, Peru, China)^13^. Midwifery education was continued in the UK, where it was integrated into hospitals, and the presence of midwifery students in clinical teams during the pandemic was considered positive^13^,^14^. As the midwifery education proceeded with distance education also in Turkey as of May 2020 in the academic year of 2019-2020, the clinical education could not take place. Considering also that midwifery students are required to attain the competencies defined as per the European Union directives, the interruption of clinical education in Turkey is a serious problem^15^. Along with the uncertainty about when formal education will be resumed, midwifery students have the risk of developing anxiety^16^,^17^.

The rapid growth in the infected cases, the feeling of uncertainty and worry created by the process, being infected with Covid-19 or having a family member infected with and/or dying of Covid-19, the education offered outside the usual way, the quarantine practice, and the economic effects also influence the students' stress and anxiety levels^6^,^12^,^16^,^18^-^20^. Moreover, hopelessness which is a problem experienced during the university years is affected by economic difficulties, career-related problems, and social relationship networks^21^. The pandemic which presents an unusual life experience can also affect hopelessness. In light of the growing concerns about the effect of Covid-19 on the mental health of vulnerable groups22, it is necessary to conduct research studies about the effect of the pandemic on university students' mental health12. In the relevant literature, there is no research study that was published about the effect of the Covid-19 pandemic on the midwifery students' mental health and e-learning. This study aims to identify the effect of the midwifery students' changing life conditions and e-learning experiences during the Covid-19 pandemic on their anxiety and hopelessness levels.

1. What level of anxiety and hopelessness do the midwifery students have?
2. Is there any statistically significant difference in the midwifery students' anxiety and hopelessness levels as per their changing life conditions?
3. Is there any statistically significant difference in the midwifery students' anxiety and hopelessness levels as per their e-learning experiences?
4. What are the factors that affect the midwifery students' anxiety and hopelessness levels?

## Material and method

### Study design

To reach all midwifery students across Turkey, the research was conducted on 25 June- 05 July 2020 through an online survey. The researchpopulation was comprised of the midwifery students in Turkey (approximately N = 10,000,000)^16^. For this cross-sectional study, the sample size was designated as a minimum of 1,067 midwifery students with 50% unknown prevalence, 3% absolute deviation, 1 design effect, and 95% confidence interval. A total of 1,320 midwifery students took part in the study. Due to missing data, 24 participants were excluded from the study and hence, the research sample was composed of 1,296 midwifery students. The research was conducted when the Turkish governmentook special measures across the country (closing down the schools and universities, making it compulsory to respect the social distance and social activities) to prevent Covid-19 from spreading and the number of cases per day which was 827 on 1 June 2020 and the number of deaths per day which was 4,563 on 1 June 2020 successively reached 1,293 and 5,167 at the end of June 2020^23^.

### Data collection

Due to restrictions stemming from the pandemic, the research data were collected via Google Forms. The survey form was created online through Google Drive. The online survey form was shared by using the Facebook, WhatsApp and Instagram accounts of the Anatolian Midwives Association Student Commission. During the research period, the announcement about the online survey was posted five times. The participant midwifery students were informed that the confidentiality of all data to be presented by them under the study would be respected. The midwifery students were required to read the consent form on the first page of the online survey form and declare that they agreed to participate in the study. Only after reading and submitting the online consent form, the participant midwifery students were allowed to fill in the survey form. Under this study, the participants were not asked to provide personal and institutional information.

### Data collection tools

The Survey Form prepared by the researchers as per the review of the relevant literature, the Generalized Anxiety Disorder Scale-7 and the Beck Hopelessness Scale were used to collect the research data.

### Survey form

The form covered the demographic variables, effects of Covid-19 on the lifestyle and distance education experiences. Through the questions about the demographic characteristics, the data about the participant students' age, class year, place of residence, and satisfaction with studying at the midwifery department were collected.

The stress factors based on the changes taking place during Covid-19 were evaluated with questions created as per the review of the relevant literature (Having Covid-19 test, being infected with Covid-19, having a family member infected with Covid-19, losing a family member due to Covid-19, having fear of the infection risk, having irregular sleep patterns, gaining weight, having any effect on the family relations, having any economic problem, needing and receiving psychological support)^6^,^12^,^16^. An overall score was obtained from 13 questions that identified the Covid-19 stressors. To make the obtained overall score more comprehensible, the overall score obtained by each participant midwifery student was converted through a scale that expressed the score out of one hundred. In this respect, the mean of overall scores obtained by the participant midwifery students was found as 37.34±14.96 points (min=0, max= 92.31). The relationship of the Covid-19 stressors with anxiety and hopelessness was examined.

The participant midwifery students' distance education experiences were addressed through questions such as which methods were used in distance education, which methods were used in the evaluation of the examinations, whether the students had access to distance education, what sort of a device the students had for accessing distance education, whether the students were motivated during distance education and whether the students attended the courses^6^,^9^,^24^,^25^.

### Beck hopelessness scale (BHS)

The scale was developed in 1974 by Beck et al. for measuring the extent of the respondent's pessimist outlook toward the future^26^. The scale was translated into Turkish in 1991 by Seber, and the validity test for the scale was performed in Turkey in 1994 by Durak and Palabıyıkoǧlu^27^,^28^. It has 20 questions. The questions are answered dichotomously as either ‘yes’ or ‘no’. The answer, ‘no’, is scored as zero in the first 11 questions whereas the answer, ‘yes’, is scored as zero in the remaining 9 questions. It is considered that, as the score obtained from the scale goes up, the hopelessness level also increases. The Cronbach Alpha coefficient was 0.93 for the scale, and it was calculated as 0.892 under this study.

### Generalized anxiety disorder scale-7 (GAD-7)

It is a 4-point Likert-type self-report scale that contained seven questions and was developed as per the criteria stipulated in the DSM-IV-TR (Diagnostic and Statistical Manual of Mental Disorders, 4^th^ Edition, Text Revision). It provides an evaluation of the generalized anxiety disorder for the last two weeks. It was adapted to Turkish by Konkan *et al.*^29^ alongside the validity and reliability test. In the ROC (Received Operating Characteristics) curve analysis conducted in the adaptation study, the cut-off point for the most acceptable specificity and sensitivity was found as 8 points for the Turkish form of the overall GAD-7. The specificity, sensitivity, positive predictive, and negative predictive values were denoted as satisfactory for this cut-off point. The results obtained from the ROC curve analysis indicates that the GAD-7 has the perfect ability to distinguish between the given classes. The Cronbach Alpha coefficient was 0.852 for the scale and it was calculated as 0.917 under this study.

### Data analysis

The dependent variables of the research were the anxiety and hopelessness. The independent variables of the study were sociodemographic features, stress factors based on the changes taking place along Covid-19 and experience and views on online education. In statistical analysis, the SPSS (Statistical Package for Social Science) was used. The mean, standard deviation, median, frequency, percentage, minimum and maximum were presented for the descriptive data. Whether the research data had normal distribution was checked with the Shapiro-Wilk Test. The data with non-normal distribution were evaluated via the Kruskal-Wallis Test and Mann-Whitney U Test. In the analysis of the data with normal distribution, Student's T-Test and one-way analysis of variance (ANOVA) were utilized. The relationship of the means of the overall scores obtained from the Covid-19 risk factors with the means of anxiety and hopelessness scale scores was analyzed through the Spearman's Rank Correlation Test. The effect of the factors associated with anxiety and hopelessness was analyzed through the enter method under the Logistic Regression Analysis, and the odds ratios (OR) were presented with a 95% confidence interval. The statistical significance was identified if the P-value was lower than 0.05 (p<0.05).

## Results

Of all participant midwifery students, 55.2% spent the pandemic period in the city center, 29.6% were first-year students and 95.2% were satisfied with being a midwifery student. It was discerned that, of all participant midwifery students, 51.7% (n=670) used smartphones in accessing the distance education, 39.4% (n=511) had no computer for distance education, 50.3% (n=652) had trouble in following up the courses due to the limited computer and internet access, 57.6% (n=746) had difficulty because of technical problems about internet connection, 63.5% failed to follow up the courses owing to the problems linked with the distance education infrastructure, 46.5% (n=602) found the instruction of theoretical courses through distance education unsatisfactory, 82.9% (n=1,074) found the instruction of practical courses through distance education unsatisfactory, and 72.5% (n=940) thought that the assignments/examinations were inadequate to measure the learning level. The mean of the participant midwifery students' anxiety scores was 8.99±5.50 points whilst the mean of their hopelessness scores was 5.93±4.99 points, and it was ascertained that 54.6% of them exhibited anxiety symptoms while 26.3% had hopelessness symptoms.

The participant students who were satisfied with studying at the department of midwifery had statistically significantly lower means of anxiety (p=0.005) and hopelessness (p=0.001) scores than those who were unsatisfied with studying at the department of midwifery (p<0.01). The students who had irregular sleep patterns, the students who gained weight, the students who were affected negatively for not being able to get out of home, the students who had tension in family relations, the students who had economic problems, the students who needed information and psychological support about anxiety/worries experienced during the Covid-19 pandemic, and the students who received information and psychological support about anxiety/worries experienced during the Covid-19 pandemic had statistically significantly higher means of anxiety and hopelessness scores (p<0.05). Moreover, it was discerned that the participant midwifery students who had the risk of being infected with Covid-19 had a statistically significantly higher mean of anxiety scores (p<0.05) (Table 1).

**Table 1:** Comparison of the means of anxiety and hopelessness scores as per the covid-19 stressors

		Anxiety				Hopelessness		
		n	Mean±Sd	Analysis	*p*	Mean±Sd	Analysis	*p*
Sociodemographic Variables								
	Province center	715	8.83±5.51	4.11	^*a*^ *0.128*	5.92±5.07	0.234	^*a*^ *0.890*
Place of residence	District	375	8.94±5.39			5.85±4.65		
	Town/village	206	9.62±5.62			6.11±5.35		
	1^st^ year	384	8.65±5.19	6.89	^*a*^ *0.075*	5.78±4.89	6.127	^*a*^ *0.106*
Class year	2^nd^ year	356	8.87±5.61			5.77±4.87		
	3^rd^ year	318	9.72±5.65			6.68±5.51		
	4^th^ year	238	8.72±5.56			5.4±4.52		
Satisfaction with the midwifery department	Yes	1234	8.89±5.47	-2.78	^*b*^ 0.005**	5.74±4.9	-5.924	^*b*^ 0.001**
**Covid-19 Stressors**	No	62	11.03±5.63			9.69±5.32		
Having Covid-19 test upon being suspicious of being infected with Covid-19	No	1253	9.02±5.51	1.14	^c^0.25	5.92±5.01	0.218	0.82
	Yes	43	8.04±4.83			6.09±4.39		
Having a family member having Covid-19 test upon being suspicious of being infected with Covid-19	No	1103	8.94±5.51	-0.68	^c^0.49	5.83±4.97	-0.55	0.12
	Yes	193	9.23±5.40			6.44±5.08		
Having Covid-19 infection	No	1103	8.98±5.52	-0.01	^c^0.98	5.92±4.99	-0.40	0.68
	Yes	23	9.00±3.84			6.34±4.82		
Having a family member with Covid-19 infection	No	1218	8.96±5.50	-0.50	^c^0.61	5.86±4.98	-0.79	0.74
	Yes	78	9.29±5.34			6.91±5.09		
Losing a relative due to the Covid-19 pandemic	No	1209	8.92±5.50	-1.59	^c^0.111	5.85±4.94	-1.89	0.05
	Yes	87	9.89±5.31	-3.93	^c^0.000	6.90±5.56	-0.01	0.99
Fearing the risk of being infected with Covid-19 during the pandemic	No	202	7.59±5.85			5.92±5.01		
	Yes	1094	9.24±5.39			5.93±4.99		
Having irregular sleep patterns during the Covid-19 pandemic	No	525	7.19±5.22	-10.03	^c^0.000	4.95±4.58	-6.03	0.00
	Yes	771	10.20±5.34			6.59±5.15		
	No	567	8.27±5.47			5.09±4.96		
Gaining weight during the Covid-19 pandemic				-4.144	^c^0.000		-0.543	0.00
	Yes	729	9.54±5.45			6.58±5.12		
Being negatively affected for not being able to get out of home during the Covid-19 pandemic	No	215	6.46±5.25	-7.52	^c^0.000	4.97±41.51	-3.08	0.00
	Yes	1081	9.49±5.40			6.12±5.06		
Having any tension in the family relations during the Covid-19 pandemic problem due to the Covid-19 pandemic	No	440	5.97±4.63	-15.99	^c^0.000	4.02±3.70	-11.42	0.00
	Yes	856	10.53±5.26			6.90±5.28		
	Yes	749	10.22±5.52			5.74±5.26		
Needing information and psychological support about anxiety/worries experienced during the Covid-19 pandemic	No	777	6.93±4.73	-18.21	^c^0.000	4.66±4.23	-11.16	**0.00**
	Yes	519	12.06±5.11			7.82±5.42		
Receiving information and psychological support about anxiety/worries experienced during the Covid-19 pandemic	No	1228	8.89±5.48	2.60	^c^0.009	5.83±4.97pad	-2.77	**0.00**
	Yes	68	10.67±5.45		^c^0.01	7.55±5.07		

a Kruskal-Wallis Test

b Mann-Whitney U Test

c Independent Samples t-test *p<0.05 **p<0.01

The mean of scores obtained by the participant midwifery students from the Covid-19 stressors had a moderately positive relationship with the mean of their anxiety scores whilst it had a relatively weak positive relationship with the mean of their hopelessness scores (Table 2).

**Table 2:** Relationships of the mean of scores of Covid-19 stressors with the mean of anxietyscores and the mean of hopelessness scores

	Mean of scores obtained from the Covid-19 stressors	

	r	*p*
**Generalized Anxiety Disorder Scale-7** **BECK Hopelessness Scale Scores**	0.472	0.001**
	0.348	0.001**

*r= Spearman's correlation coefficient*

** *p<0.01*

There was a statistically significant difference in the means of anxiety scores as per the device type used by the participant midwifery students for distance education (p=0.001), and the students who used computers had lower means of anxiety (p=0.001) and hopelessness (p=0.002) scores than those using smartphones (p<0.01). The students who had trouble in following up the courses due to the limited computer and internet access had statistically significantly higher means of anxiety (p=0.001) and hopelessness (p=0.001) scores than those who had no trouble in this sense (p<0.01). Likewise, the students who had difficulty in following up the courses through the distance education system due to the technical problems about the internet connection had statistically significantly higher means of anxiety (p=0.001) and hopelessness (p=0.001) scores than those who had no difficulty in this regard (p<0.01). The students who found that the instruction of the theoretical courses through distance education was inefficient had statistically significantly higher means of anxiety (p=0.001) and hopelessness (p=0.001) scores than those who found such theoretical instruction efficient and partially efficient (p<0.05). The students who found that the instruction of the practical courses through distance education was inefficient had a statistically significantly higher mean of anxiety scores than those who found such practical instruction efficient and partially efficient (p=0.007, p=0.001, p<0.01). The students who found that the instruction of the practical courses through distance education was partially efficient had a statistically significantly lower mean of anxiety scores than those who found such practical instruction inefficient (p=0.007, p<0.01). The students who thought that the assignments/examinations utilized under the distance education system were adequate for measuring the student learning had statistically significantly lower means of anxiety (p=0.001) and hopelessness (p=0.001) scores than those who thought otherwise (p<0.01). The students who attained their targeted academic achievements well beyond their expectations in the process of distance education had statistically significantly lower means of anxiety (p=0.001) and hopelessness (p=0.001) scores than those who attained academic achievements at the expected level (p<0.01). The students who suffered the loss of motivation for studying during the distance education had statistically significantly higher means of anxiety (p=0.001) and hopelessness (p=0.001) scores than those who did not lose motivation (p<0.01) (Table 3).

**Tablo 3:** Comparison of the means of anxiety and hopelessness scores as per the students'distance education experiences

		Anxiety		Hopelessness	

		Mean±Sd	Analysis	Mean±S	Analysis
			p	d	p
Device used for accessing distance education	Computer	8.28±5.34	χ^2^:19.995	5.48±4.84	χ^2^:11.913
	Tablet	9.32±4.69	^a^0.001**	4.84±4.11	^a^0.003**
	Smartphone	9.62±5.59		6.37±5.12	

Having trouble in following up the courses due to limited computer and internet access	Yes	10.16±5.52	Z:-7.749	6.77±5.24	Z:-6.181
	No	7.83±5.23	^b^0.001**	5.1±4.6	^b^0.001**

Having difficulty in following up the courses due to technical problems about internet connection	Yes	10.16±5.41	Z:-9.146	6.8±5.23	Z:-7.415
	No	7.4±5.22	^b^0.001**	4.75±4.39	^b^0.001**

Finding the instruction of the theoretical courses through distance education efficient	Efficient^1^ Partially	6.59±5.47	χ^2^:62.771	4.81±4.57	χ^2^:27.642
	efficient^2^	8.45±5.23	^a^0.001**	5.44±4.77	^a^0.001**
	Inefficient^3^	10.07±5.49		6.65±5.19	

Finding the instruction of the practical courses through distance education efficient	Efficient^1^			5.42±4.58	χ^2^:9.604
		7.38±6.31	χ^2^:37.690	4.82±4.31	^a^0.008**
	Partially efficient^2^	6.92±5.17	^a^0.001**	6.13±5.09	
	Inefficient^3^	9.39±5.42			

Thinking that the assignments / examinations used under distance education system are adequate for measuring the student learning	Yes	7.94±5.46	Z:-4.434	5.2±5.4.74	Z:-3.514
	No	9.39±5.46	^b^0.001**	6.21±5.5.06	^b^0.001**

Attaining the targeted academic achievements in the process of distance education	Beyond expectations	8.01±5.16	Z:-7.312	5.05±5.4.53	Z:-7.427
	At the expected level	10.37±5.67	^b^0.001**	7.17±5.5.35	^b^0.001**

Experiencing loss of motivation for studying	Yes	9.52±5.42	Z:-7.094	6.22±5.08	Z:-4.160
	No	6.98±5.32	^b^0.001**	4.84±4.5	^b^0.001**

Upon the review of the relationship of the behaviors, which are performed for coping with stress during the process of the pandemic in a fashion different from those in daily life, with anxiety and hopelessness, it is discerned that the students who developed/started hobbies (p=0.001) and the students who read books (p=0.020) had statistically significantly lower means of anxiety scores (p<0.01, p<0.05). Moreover, the students who prayed (p=0.034), the students who played sports (p=0.002), the students who developed/started hobbies (p=0.001), and the students who read books (p=0.001) had statistically significantly lower means of hopelessness scores (respectively p<0.05, p<0.01, p<0.01, p<0.01) (Table 4).

**Table 4:** Comparison of the means of anxiety and hopelessness scores as per the coping behaviors

Behaviors performed for coping with stress during the process of pandemic in a fashion different from the normal behaviors in the daily life		Anxiety		Hopelessness	

		Mean±S	Analysis	Mean±S	Analysis
		d	p	d	p
Praying	Yes	8.95±5.44	Z: -0.124	5.62±4.78	Z: -2.122
	No	9.04±5.57	^***b***^ ***0.901***	6.32±5.24	^***b***^ ***0.034*** *

Playing sports	Yes	8.59±5.26	Z: -1.928	5.38±4.69	Z: -3.140
	No	9.26±5.64	^***b***^ ***0.054***	6.3±5.16	^***b***^ ***0.002*** **

Having video calls with friends	Yes	8.82±5.28	Z: -0.918	5.79±4.91	Z: -1.135
	No	9.3±5.87	^***b***^ ***0.359***	6.18±5.14	^***b***^ ***0.256***

Developing/starting hobbies	Yes	8.14±5.26	Z: -5.481	5.09±4.64	Z: -6.425
	No	9.85±5.6	^***b***^ ***0.001*** **	6.78±5.2	^***b***^ ***0.001*** **

Reading books	Yes	8.68±5.36	Z: -2.328	5.45±4.71	Z: -4.058
	No	9.48±5.68	^***b***^ ***0.020*** *	6.68±5.33	^***b***^ ***0.001*** **

b *Mann-Whitney U Test*

* *p<0.05*

** **p<0.01

It was found that having trouble in following up the courses, finding the instruction of the theoretical courses through distance education partially efficient, finding the instruction of the theoretical courses through distance education inefficient, fearing the risk of being infected with Covid-19, having irregular sleep patterns, having tension in family relations, having economic problems, receiving psychological support about anxiety and worries, and having hopelessness increased the anxiety score respectively by 1.438 times (CI:1.103-1.875), 1.976 times (CI:1.262-3.094), 2.158 times (1.376-3.386), 1.601 times (CI:1.118-2.292), 1.694 times (CI:1.301-2.205), 2.362 times (CI:1.780-3.134), 1.434 times (CI:1.095-1.879), 2.914 times (CI:2.208-3.8477), and 2.878 times (CI:2.075-3.991) (p<0.05). Having trouble in following up the courses, losing the motivation for studying, gaining weight, having the negative effect of not going out of home, having tension in family relations, feeling the need to have psychological support, receiving psychological support about anxiety and worries, and having anxiety increased the hopelessness score consecutively by 1.980 times (CI:1.459-2.687), 1.460 times (0.969-2.200), 1.669 times (CI:1.252-2.226), 0.550 times (CI:0.359-0.844), 1.789 times (CI:1.235-2.594), 2.006 times (CI:1.488-2.703), 1.875 times (CI:1.083-3.247), and 2.755 times (CI:1.985-3.823) (p>0.05) (Table 5). The analysis model developed for both logistic regressions are statistically significant and the predictive coefficients are at a high level for anxiety (71.7%) and hopelessness (73.8%) (Table 5).

**Table 5:** Regression analysis of the factors affecting anxiety and hopelessness

Anxiety		*p*	OR	95% C.I.	
				Lower	Upper
	Having trouble in following up the courses (1)	0.007**	1.438	1.103	1.875
	Finding the instruction of the theoreticalcourse through distance education	0.003**			
	Partially efficient	0.003**	1.976	1.262	3.094
	Inefficient	0.001**	2.158	1.376	3.386
	Fearing the risk of being infected with Covid-19 (1)	0.010*	1.601	1.118	2.292
	Having irregular sleep patterns (1)	0.000**	1.694	1.301	2.205
	Having tension in family relations (1)	0.000**	2.362	1.780	3.134
	Having economic problems (1)	0.009**	1.434	1.095	1.879
	Receiving psychological support about anxiety/worries (1)	0.000**	2.914	2.208	3.847
	Having hopelessness	0.000**	2.878	2.075	3.991

**Hopelessness**					

	Having trouble in following up the courses (1)	0.000**	1.980	1.459	2.687
	Losing the motivation for studying (1)	***0.070***	1.460	0.969	2.200
	Gaining weight (1)	0.000**	1.669	1.252	2.226
	Having the negative effect of not going out of home (1)	0.006**	0.550	0.359	0.844
	Having tension in family relations (1)	0.002**	1.789	1.235	2.594
	Feeling the need to have psychological support (1)	0.000**	2.006	1.488	2.703
	Receiving psychological support about anxiety/worries (1)	0.025*	1.875	1.083	3.247
	Anxiety	0.000**	2.755	1.985	3.823

* *p<0.05*

** *p<0.01*

## Discussion

Identifying how the midwifery students were affected by the changing living conditions due to the pandemic, the effects of the changing conditions on their education, and also the implications of this situation on their anxiety and hopelessness levels is important to the management and planning of education. This is the first study to investigate the impact of midwifery students' changing living conditions and e-learning experiences on anxiety and hopelessness. Collecting data online with self-reporting questionnaires is considered as a limitation of the study. Nevertheless, the strength of this study is that it is the first study to reveal online education, changing living conditions, anxiety and hopelessness during the pandemic process. Another strength of the study is that the sample represents all midwifery students in Turkey. Determining the processes experienced by midwifery students during the covid 19 period will shed light on the realization of the global solutions. The discussion is written in the context of the study questions.

The study, it was found that the participant midwifery students had a high prevalence of generalized anxiety during the Covid-19 pandemic, and the state of their hopelessness was also affected by the pandemic. The anxiety level was affected by factors such as experiencing economic problems, having the fear of being infected with Covid-19, having sleep disorder, and finding the instruction of theoretical courses through distance education inefficient/partially efficient. It was ascertained that the motivation loss, weight gaining, being unable to leave home, and needing psychological support affected the hopelessness level. Moreover, it was discerned that receiving psychological support, having trouble in following up the courses, and having tension in family relations affected the anxiety and hopelessness levels, and anxiety and hopelessness affected each other. The discussion took place in light of these findings under the study.

In this study, 54.6% of the participant midwifery students exhibited anxiety symptoms. In a study, it was put forward that 5.5% of the midwifery students had medium and high anxiety levels (Beck Anxiety Inventory) (n=972) when the first Covid-19 case was identified in Turkey in March 2020^16^; on the other hand, in another study performed on the university students in June 2020 (n=358), it was setforth that 52% of the students had medium and high anxiety levels (GAD-7)^30^. It was identified that 48.2% of the university students had anxiety due to the Covid-19 pandemic as per a study in Brazil, 42.8% of the nursing students had medium level anxiety and 13.1% of the nursing students had severe anxiety as per a study in Israel (GAD-7), and 42% of the university students exhibited stress, anxiety and depression symptoms as per a study in India^24^,^31^-^33^. In a study conducted recently in China (n=746,217), it is stated that the mental problems were quite prevalent among the university students, and 34.9%, 21.1%, and 11,0% of the university students successively exhibited acute stress, depression, and anxiety symptoms (GAD-7)^34^. It is considered that the reason for the participant midwifery students to have high-level anxiety within the scope of the current study is that the study was performed during the late stages of pandemic and the uncertainty of the process still continued.

The social and economic problems, deficiencies in education, and worries about the future trigger the feeling of hopelessness in the students^35^. In this current study, it was found that more than one in four participant midwifery students (26.3%) exhibited hopelessness symptoms. It is asserted that approximately one in three participants in the general population exhibited moderate and severe hopelessness symptoms during the process of Covid-19^36^. The effect on the hopelessness level seems inevitable in the period of the pandemic when extraordinary circumstances are experienced. In this current study, the students' anxiety levels were affected by factors such as having the risk of being infected with Covid-19, staying at home during the quarantine, having irregular sleep patterns, having tension in family relations, receiving psychological support, and finding th instruction of the theoretical courses through distance education inefficient and partially efficient. In a study^30^, it was demonstrated that the negative effect of Covid-19 on education, economy, and relations affected the perceived stress in university students. In the study by Kartal and Kaykısız^37^, it was found that 84.8% of the midwifery students had changes in their sleep patterns during the process of Covid-19.

In another study by Zeng *et al.*^38^, it was stated that the nursing students' mental health problems were associated with low sleep quality and negative life experiences. Another factor with an effect on the anxiety during the pandemic is the apprehension about being infected with Covid-19. Likewise, in the studies performed on the nursing students, it is put forth that worrying about being infected with Covid-19^30^,^39^ and having an acquaintance testing positive for Covid-19 raised the nursing students' anxiety and stress levels^30^.

Along with the social isolation and quarantine processes which accompanied the Covid-19 pandemic, the individuals began to spend more time at home with their families. In this current study, it is noted that the family relations were strained and this in return increased anxiety and hopelessness. Even if social isolation is an effective method for controlling the infection, it increases the violence in the family as it is accompanied by serious social, psychological, economic, and societal consequences 40,41. Therefore, while addressing the psychological aspects of the pandemic, it is important to evaluate the problems such as the violence in the family and take steps to protect the population at risk.

The quarantine leads to social and economic consequences^4^. As it is discerned in this current study, receiving/needing psychological support affects generalized anxiety and hopelessness. The Covid-19 pandemic has the risk of interrupting he delivery of mental health services. The social and economic consequences which accompany the quarantine raise the general barriers inhibiting the search for care^42^. In this context, providing mental health services compatible with the quarantine conditions and offering easy access to these services will be of use.

In distance education, which is different from the usual formal education format, not having communication as in face-to-face education can make the students feel anxious. In this current study, it is discerned that the participant midwifery students who did not find the instruction of the theoretical courses through distance education efficient had higher levels of anxiety. Likewise, in a study, it is stated that the learning of 44% of the students was ‘worsened a little’ and the learning of 26% of the students was ‘worsened significantly’^43^, and in another study, it was set forward that 77.4% of the students perceived the distance education negatively and the distance education had very little effect on the learning of 86% of the students^25^. It is considered that having an education that equips the students with professional qualifications through distance learning in a vital area like healthcare and its difference from the experience of face-to-face learning that was adopted up to present raised the anxiety and hopelessness levels.

In the distance education activities during the pandemic, certain setbacks come into play due to the technical infrastructure and connection problems. As per this current study, anxiety and hopelessness levels of the students who have difficulty in following up the courses due to the technical connection problems increase. It is discerned that 57.6% of the students in this current study, all students in the study by Kürtüncü and Kurt9, and 53.9% of the students in the study by Keskin and Özer^44^ were faced with technical problems. In a study performed in India, it is put forward that 32.4% of the students had problems with the internet connection^32^. In a study carried out in Pakistan, it was set forth that the main challenges faced by the university students were the shortcomings in accessing internet facilities, lack of proper interaction and communication with the students and instructors, and ineffective technology^45^. In the study by Subedi *et al.*^46^, it was stated that 63.2% of the students had trouble with the online classrooms due to the power outages, 63.6% experienced internet problems, and 64.3% used extra internet for distance education^46^. The findings of this current study are interpreted that the digital separation was a significant problem and raised the anxiety and apprehension levels in Turkey as in the case of developing countries.

According to the findings of this current study, the loss of motivation for studying affects the midwifery students' hopelessness levels. In the study by Wang *et al.*^8^, it was found that the students' burnout levels increased, and also the attendance to the courses and the permanence of learning decreased. In the study by Peloso *et al.*^31^, 33.4% of the students reported that having a study routine and getting motivation were the biggest challenges. This situation may be arising due to the circumstances such as not being able to be present in the campus and classroom setting and being devoid of having interactions with peers. It is observed that people under intense stress have emotional eating behavior in particular^47^. The increasing stress linked with the uncertainty of the Covid-19 process, being quarantined, and being infected are associated with the weight gain by the adults during the current pandemic48. Identified within the scope of this current study as a factor increasing hopelessness, gaining weight is considered to be associated with the physical and psychological effects of the quarantine practice.

The finding of this current study that the anxiety and hopelessness affected each other significantly is compatible with the previous studies that analyzed the psychological effects of the pandemic 36,49. During the Covid-19 pandemic, psychological support should be provided as much as medical support. As the increase in anxiety and hopelessness levels is likely to pave the way for unintended consequences such as suicidal ideation, it is essential to identify the risky circumstances and apply the necessary interventions^50^. In this context, while the quarantine is used for preventing the infection from spreading, the effects of social isolation and loneliness should not be neglected.

## Conclusion

In this study, it was ascertained that more than half of the participant midwifery students had anxiety symptoms and approximately one in four participant midwifery students exhibited hopelessness symptoms. The life habits changing alongside the Covid-19 process and the experiences related to distance education affect anxiety and hopelessness.

As well as the Covid-19 pandemic, health, social life and economic changes and the digital separation which accompanies distance education affects mental health. Solving the technical problems in distance education, facilitating the follow-up of the courses, increasing the number of interactive courses, and supporting the students' behaviors such as developing hobbies, reading books, and doing sports during the pandemic will be useful in the management of the process. To reduce the hopelessness and anxiety levels, it is also essential to equip the midwifery students with problem-solving and coping skills. In this context, it is recommended that easy access to mental health support services be provided. Moreover, under the prospective research, performing longitudinal studies about how these psychological effects progressed is recommended.

### Strengths and limitations

Due to restrictions stemming from the pandemic, the research data were collected via Google Forms. Collecting data online with self-reporting questionnaires is seen as a limitation of the study.

On the other hand, the strength of this study is that it is the first study to reveal the effects of the changing life conditions, e-learning experiences on anxiety and hopelessness of midwifery students during the pandemic process. Another strength of the study is that the sample represents all midwifery students in Turkey.

## Recommendations

### Short term recommendations

Measures should be taken to facilitate the distance education process for students. Removing the access barrier due to the restricted internet package of the students and providing internet package support, facilitating course follow-up in distance education, increasing the number of lessons with icebreakers or digital interactions such as surveys, video interpretations, case studies, mini quizzes used in lessons, supporting educational activities by giving students homework after the lesson and following these homeworks and instilling a sense of responsibility in students are among the constructive suggestions that facilitate this process.

In addition, supporting students with social interaction during the pandemic period, informing and encouraging access to psychosocial support offered by mental health professionals, and the existence of behaviors and opportunities such as taking up hobbies, reading books, and doing sports even by participating in online courses will have a positive effect on managing the process.

### Medium term recommendations

In order to reduce hopelessness and anxiety levels, it is also necessary to provide midwifery students with problem solving and coping skills. In this context, it is recommended to facilitate access to mental health support services. To facilitate this access, when students return to formal education, cooperation with health services units of universities may be one of the possible alternatives, which may increase the psychosocial well-being of students. At the same time, the gradual return to formal education, the measures taken to reduce the risk of transmission and knowing the centers to which they can apply in suspicious cases can also reduce the anxiety and hopelessness levels of the students regarding the formal education process.

In addition, considering the possible problems that students may experience in their integration into formal education and clinical practices after the education process they spend with constant screen exposure, academics should create action plans and strategic plans in cooperation with all relevant official institutions at the country level.

### Long term goals

In order to avoid similar problems when faced with any humanitarian crisis that may occur in the future, apart from Covid-19, it is very important to consider the results of systematic compilation and meta-analysis based on studies on education in all humanitarian crises and during the Covid 19 process. Long-term educational goals should include solving infrastructure problems and the existence of systems with extensive access capabilities. In addition, long-term studies on how the psychological effects experienced in the pandemic progress can be counted among the long-term recommendations.
